# Excited-State Dynamics in Colloidal Semiconductor Nanocrystals

**DOI:** 10.1007/s41061-016-0060-0

**Published:** 2016-08-09

**Authors:** Freddy T. Rabouw, Celso de Mello Donega

**Affiliations:** 1 Inorganic Chemistry and Catalysis, Debye Institute for Nanomaterials Science, Utrecht University, PO Box 80000, 3508 TA Utrecht, The Netherlands; 2 Soft Condensed Matter, Debye Institute for Nanomaterials Science, Utrecht University, PO Box 80000, 3508 TA Utrecht, The Netherlands; 3 Present Address: Optical Materials Engineering Laboratory, ETH Zurich, 8092 Zurich, Switzerland; 4 Condensed Matter and Interfaces, Debye Institute for Nanomaterials Science, Utrecht University, PO Box 80000, 3508 TA Utrecht, The Netherlands

**Keywords:** Semiconductor nanocrystals, Colloids, Exciton dynamics, Nanoscale, Auger relaxation

## Abstract

Colloidal semiconductor nanocrystals have attracted continuous worldwide interest over the last three decades owing to their remarkable and unique size- and shape-, dependent properties. The colloidal nature of these nanomaterials allows one to take full advantage of nanoscale effects to tailor their optoelectronic and physical–chemical properties, yielding materials that combine size-, shape-, and composition-dependent properties with easy surface manipulation and solution processing. These features have turned the study of colloidal semiconductor nanocrystals into a dynamic and multidisciplinary research field, with fascinating fundamental challenges and dazzling application prospects. This review focuses on the excited-state dynamics in these intriguing nanomaterials, covering a range of different relaxation mechanisms that span over 15 orders of magnitude, from a few femtoseconds to a few seconds after photoexcitation. In addition to reviewing the state of the art and highlighting the essential concepts in the field, we also discuss the relevance of the different relaxation processes to a number of potential applications, such as photovoltaics and LEDs. The fundamental physical and chemical principles needed to control and understand the properties of colloidal semiconductor nanocrystals are also addressed.

## Introduction

Since the pioneering work of Brus, Ekimov, and many others in the early 1980–1990s [1–13], the study of semiconductor nanocrystals (NCs) has developed into a mature, dynamic and multidisciplinary research field, which attracts increasing attention worldwide, both for its fundamental challenges and its potential for a number of technologies (light emitting devices, solar cells, luminescent solar concentrators, optoelectronics, sensing, thermoelectrics, biomedical applications, catalysis) [14–32]. Colloidal semiconductor NCs are particularly attractive, since they consist of an inorganic core that is coated with a stabilizing layer of (usually) organic ligand molecules. This hybrid inorganic–organic nature makes them very versatile nanomaterials that combine size-, shape-, and composition-dependent optoelectronic properties of the core with easy surface manipulation and solution processing [16].

Here, we will address the excited-state dynamics in colloidal semiconductor NCs, covering a time scale that spans over 15 orders of magnitude, from a few femtoseconds to seconds after photoexcitation. We intend to provide a critical overview of the field, in which the recent advances are discussed and the outstanding challenges are identified. The relevance of different excited-state relaxation processes to a number of potential applications will also be highlighted. This review is not meant to be exhaustive, but rather to convey a concise account of the state-of-the-art, in which the essential aspects are outlined and discussed. For further details or an in-depth treatment of topics that are beyond the scope of this work, the reader will be referred to the recent literature. This article is organized as follows. In Sect. 2, we discuss how excitons in semiconductor NCs are affected by nanoscale effects. In Sect. 3, the relaxation dynamics of nanoscale excitons in colloidal semiconductor NCs is addressed, with particular emphasis on the processes that occur at different time scales after photoexcitation. In the last section, we summarize the essential aspects discussed and the outlook for the field.

## Excitons in Semiconductor Nanocrystals

### Quantum Confinement Effects: Squeezing and Shaping Nanoscale Excitons

**Fig. 1:**
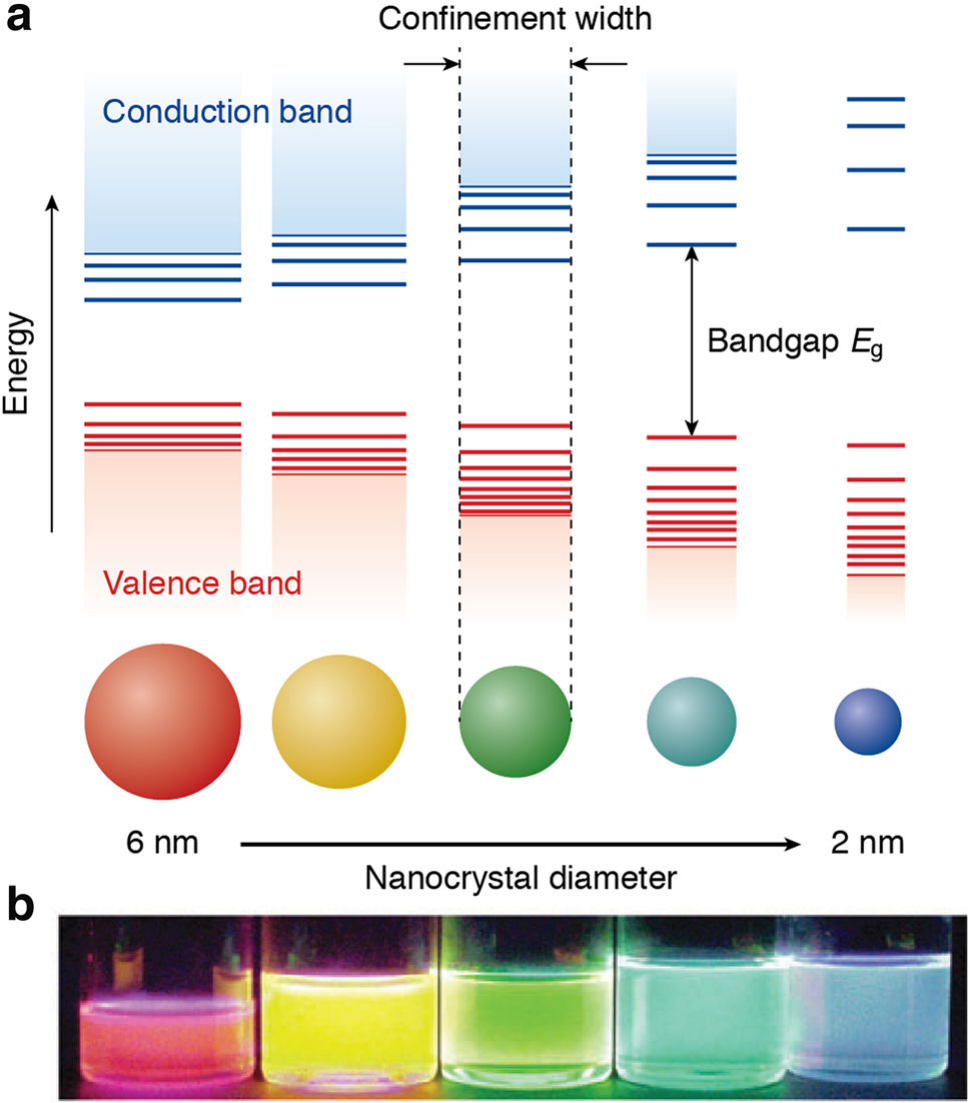
**a** Schematic representation of the quantum confinement effects: the bandgap (or HOMO–LUMO gap) of the semiconductor nanocrystal increases with decreasing size, while discrete energy levels arise at the band-edges. The energy separation between the band-edge levels also increases with decreasing size. **b** Photograph of five colloidal dispersions of CdSe QDs with different sizes, under excitation with a UV-lamp in the dark. The color of the photoluminescence changes from red to blue as the QD diameter is reduced from 6 to 2 nm. Adapted from Ref. [16] with permission of the Royal Society of Chemistry

**Fig. 2:**
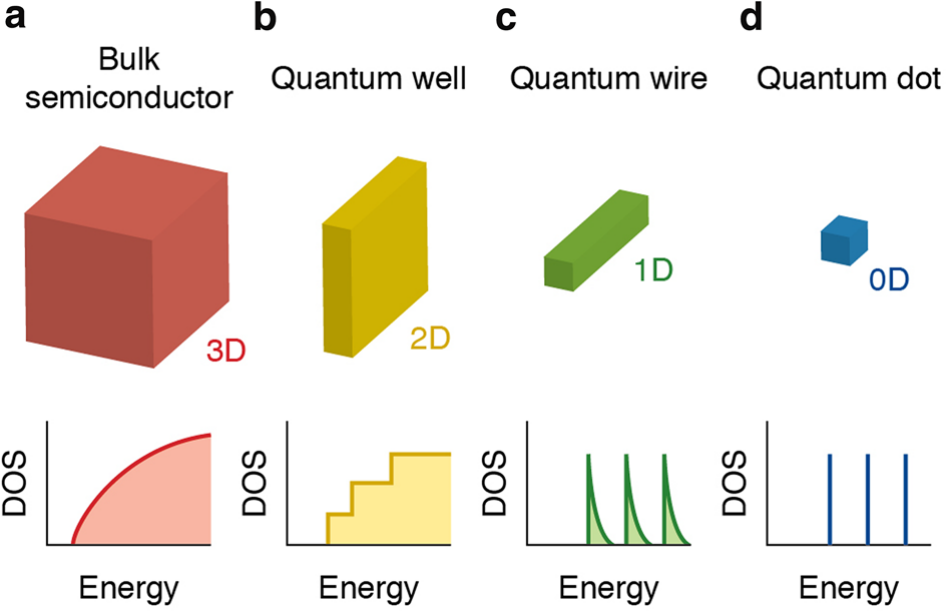
Schematic illustration of the energy level structure of a bulk semiconductor (**a**), and semiconductor nanostructures (**b**–**d**) with reduced dimensionality. **b** 2D semiconductor nanostructure or quantum well. **c** 1D semiconductor nanostructure or quantum wire. **d** 0D semiconductor nanostructure or quantum dot.* DOS* represents the density of electronic states

Absorption by a semiconductor of a photon with energy equal to or larger than its bandgap (*E*_g_) promotes an electron from the valence band (VB) to the conduction band (CB), leaving a hole in the VB, and forming an exciton (i.e., an electron–hole pair bound by Coulomb interaction). The impact of spatial confinement to the nanoscale depends on characteristic length scales associated with the physical property under consideration. In the case of the properties of excitons in semiconductors, this characteristic length scale is given by the exciton Bohr radius (*a*_0_), which ranges from ~2 to ~50 nm, depending on the material [33]. For semiconductor NC sizes of approximately *a*_0_ and smaller, the exciton wave function is affected by spatial confinement [33]. This induces size-dependent changes in the density of electronic states and in the energy separation between them, which are manifested in an increase of the bandgap (or HOMO–LUMO energy gap) and the appearance of discrete energy levels near the band edges with decreasing NC dimensions (Fig. 1) [33–35]. This effect is commonly referred to as quantum confinement, and makes it possible to tune the optical spectra (absorption and photoluminescence, PL) of semiconductor NCs through a wide spectral window by simply changing their size, while keeping their composition constant (Fig. 1). Further, the degree of quantum confinement may be different in different directions of the NC, depending on its size and shape (Fig. 2) [33, 35]. If the exciton is spatially confined in all directions, a quantum dot (QD) is obtained, while NCs in which the exciton is confined only in the diameter direction are referred to as quantum wires. Quantum confinement in the thickness direction only (2D confinement) results in a quantum well. Quantum rods are NCs in transition from the zero-dimensional confinement regime of QDs to the 1D confinement regime of quantum wires. This makes the optoelectronic properties of semiconductor NCs also strongly shape-dependent. In the quantum confinement regime, the size and shape of semiconductor NCs also have an impact on the exciton fine-structure. The exciton fine-structure is the way in which the energy states of the exciton are split by effects of the crystal field asymmetry, NC shape anisotropy, and electron–hole exchange interaction [36–38]. Exciton fine-structure splitting is analogous to singlet–triplet splitting in organic molecules, but the energy splittings for an exciton in a NC are typically smaller, namely only a few meV. Effects of the exciton fine-structure are therefore relevant only at low temperatures (below 100 K), where they affect the temperature- and magnetic-field dependences of the exciton lifetimes. These effects are beyond the scope of this review. The interested reader is referred to a number of publications addressing this topic in detail [36–48].

Phonons (i.e., lattice vibrations) have a pervasive role in semiconductors, and therefore coupling of charge carriers and excitons to phonons plays a decisive role in a wide range of properties [49]. The interaction between phonons and excitons in nanoscale semiconductors is expected to differ from that in bulk materials due to both quantum confinement effects on the exciton energy levels and dimensional confinement of phonon modes (i.e., the phonon wavelength cannot be larger than the NC size) [49]. Coupling of photogenerated carriers to phonons provides an important energy relaxation pathway, thus being essential to a number of photophysical processes in semiconductor NCs (e.g., exciton relaxation dynamics, carrier cooling, thermal transport) [42, 50–53]. Moreover, coupling to acoustic phonon modes determines the homogeneous linewidths of optical transitions [54, 55], while coupling to optical phonon modes has been observed to relax selection rules at low temperatures, yielding distinct phonon-assisted transitions (the so-called phonon replicas) [56, 57].

### Composition Effects: Tailoring the Property Gamut

As mentioned in Sect. 2.1 above, the exciton Bohr radius is a material property. As a result, different semiconductors experience quantum confinement at different NC sizes, depending on their exciton Bohr radius. Moreover, the bulk bandgap of different semiconductor materials covers a range of energies from the infrared to the ultraviolet. As a result, the bandgap of different semiconductor NCs is tunable over different spectral windows [16, 26, 33, 35]. For example, the lowest energy absorption transition of CdSe QDs can be tuned from 1.75 eV (the bulk *E*_g_ value) to 2.65 eV for diameters ranging from ~10 nm (*a*_0_ = 4.9 nm) to 2 nm [58], while that of PbSe QDs can be tuned from 0.3 eV (the bulk *E*_g_ value) to 1.5 eV for diameters ranging from ~100 nm (*a*_0_ = 46 nm) to 2 nm [59]. The optoelectronic properties of semiconductor NCs can thus be tailored by choosing their composition and controlling their size and shape.

**Fig. 3:**
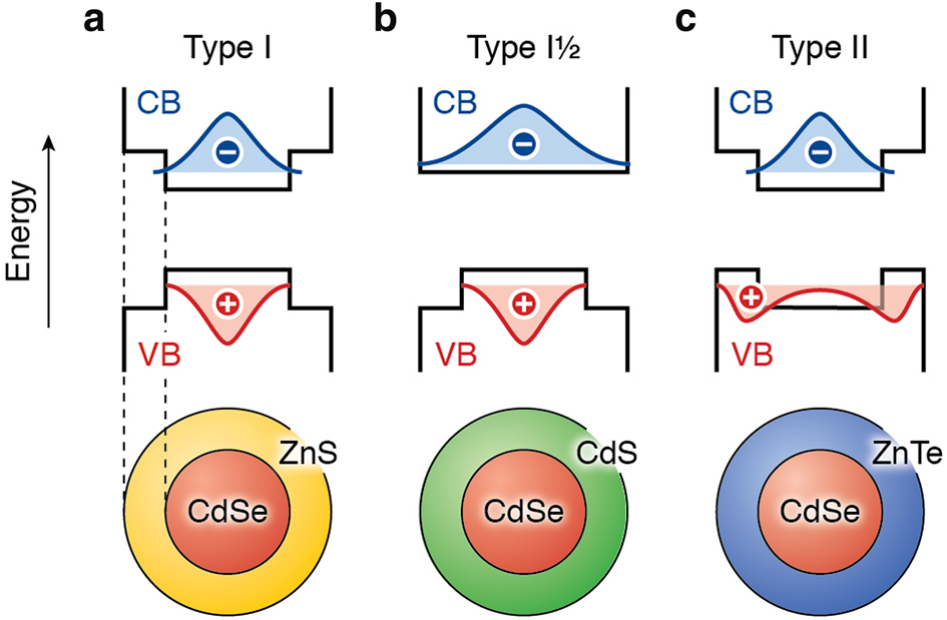
Schematic representation of the three limiting charge carrier localization regimes in core/shell semiconductor hetero-NCs. The energy of the bulk conduction and valence band edges (CB and VB; *black solid lines*) sets the potential energy of the charge carriers, while the effective mass from the bulk band structure determines the kinetic energy. The wave functions of the lowest-energy electron (*blue*) and hole (*red*) states are schematically depicted. The charge carriers tend to localize in the part of the hetero-NC with the lowest potential energy. **a** In type-I hetero-NCs, such as CdSe/ZnS, both charge carriers co-localize in the same part. **b** In type-I½ hetero-NCs, such as CdSe/CdS, one charge carrier delocalizes over the entire NC while the other one is localized in one part. **c** In type-II hetero-NCs, such as CdSe/ZnTe, the two charge carriers are spatially separated, each in a different part, forming a spatially indirect exciton

The control over the properties of colloidal NCs can be extended further by using NCs consisting of two (or more) different semiconductors joined together by heterointerfaces, i.e., hetero-NCs [16]. The spatial localization of the photogenerated charge carriers in hetero-NCs can be manipulated by controlling the band offsets of the materials that are combined at the heterointerface (Fig. 3) [16]. In type-I hetero-NCs both carriers are confined in the same material (e.g., CdSe/ZnS, InP/ZnS). In contrast, in type-II hetero-NCs a spatially indirect exciton is formed, as the electron and hole wave functions are centered in different materials, and thus in different segments of the hetero-NC (e.g., CdSe/ZnTe, CdSe/CdTe). In type-I^1/2^ (or quasi-type-II) hetero-NCs one carrier is delocalized over the whole volume of the hetero-NC, while the other is localized in one of the segments (e.g., CdSe/CdS, ZnSe/CdSe). This allows the electron–hole spatial overlap to be tailored by controlling the size, shape, and composition of each segment of the hetero-NC, which has a dramatic impact on several properties (viz., quantum yields, stability, PL wavelength [15, 16, 21, 60, 61], reabsorption cross section [22, 29, 62–64], radiative lifetimes [60, 64–66], exciton-phonon coupling strength [67–69], Auger recombination [66, 70–72], hot carrier relaxation [51, 73], thermal quenching [74, 75]). The general trend is that the exciton lifetime, exciton-phonon coupling, and PL wavelength increase when going from the type-I to the type-II localization regimes, while Auger recombination rates and hot carrier relaxation rates are reduced. This is beneficial not only for technologies relying on efficient charge separation, such as solar cells, photodetectors, and photocatalysis, but also for applications requiring light emission, such as luminescent solar concentrators (reduced reabsorption losses) [22, 29] and lasers (lower lasing threshold) [14]. However, short lifetimes and narrow bandwidths are preferred for application in LEDs, since this increases the output saturation threshold and the color-rendering index [19]. The electron–hole wave function overlap in hetero-NCs has also been shown to affect the exciton fine-structure [76–79].

The properties of colloidal semiconductor hetero-NCs can also be tuned under constant size, shape, and total composition by making use of controlled interdiffusion. The elemental distribution profile of hetero-NCs can go from a core/shell geometry with a sharp heterointerface to a homogenous alloy QD, via gradient alloy NCs of increasing homogeneity which seamlessly connect these two extremes [80–82]. In this way, the optoelectronic properties can be continuously tuned from those of core/shell hetero-NCs (type-I, type-II or type-I^1/2^) to those of homogeneous alloy NCs, with preservation of the total volume and composition of the NC [80–83]. Moreover, core/shell hetero-NCs with a gradient alloy heterointerface have been shown to possess unique properties, such as reduced Auger recombination rates and lower threshold for amplified spontaneous emission [84]. Alloy QDs and graded interface core/shell hetero-NCs can also be directly synthesized and have attracted increasing interest in the last few years, leading to the investigation of several II-VI and IV-VI compositions [viz., Cd(Te,Se), Cd(S,Se), Pb(S,Se), (Cd,Zn)Se, (Cd,Zn)S, (Cd,Zn)(S,Se)] [80–89].

Another effective strategy to impart novel properties (e.g., optical or magnetic) to semiconductor NCs is the intentional introduction of impurities (doping) [90]. Doping of bulk materials is a very well developed field, which underpins most of our present technologies, since the properties of materials for lighting, electronic and optoelectronic applications are largely controlled by dopants. In contrast, the precise doping of NCs is still an underdeveloped field, which is however booming and has delivered great successes and many novel materials in recent years [28, 91–100].

Over the last few years, potential toxicity and environmental impact have become important driving forces in the quest for novel semiconductor NCs and hetero-NCs [32], since the best-developed systems to date are based on Cd- and Pb-chalcogenides. A remarkable degree of control over size, shape and composition has been achieved for these types of NCs [14–16, 19, 21, 26, 28, 61], but widespread deployment into consumer products is severely limited by toxicity concerns. This has motivated an increasing research effort on alternative compositions that are based on less toxic elements, such as copper chalcogenides (e.g., CuInS_2_) [24, 26, 32], InP [26, 101], and Si [102].

### Nanoscale Surfaces: far from “Superficial”

The most prevalent nanoscale effect is the increase in the surface-to-volume ratio with decreasing size. Surface atoms comprise only a very small fraction of the constituents of bulk solids, and therefore have a negligible contribution to the material properties. In contrast, the fraction of atoms at surfaces and/or (hetero) interfaces is significant at the nanoscale and becomes increasingly larger as the NC dimensions are further reduced. As a result, the contribution of the surface atoms to the properties of the NC becomes increasingly larger, eventually giving rise to completely novel properties. Surface atoms have fewer neighbors, and therefore possess a higher free energy and unsatisfied chemical bonds (the so-called dangling bonds). The increasingly larger surface/volume ratio of NCs will thus render them more reactive and dynamic than bulk crystals, which impacts a number of properties, such as melting temperatures, solubility, plasticity, catalytic activity, crystal structure, and colloidal dispersibility [16, 103].

The NC surface is a dynamic interface between the inorganic semiconductor core and the ligand shell. The interaction between the semiconductor core and the ligands is crucially relevant during the synthesis of colloidal NCs, since it affects both the thermodynamics and kinetics of their nucleation and growth [16]. It is thus largely responsible for the remarkable degree of control achieved over the size, shape, and composition of semiconductor NCs and hetero-NCs [16]. Another important consequence of the large contribution of surface atoms to the properties of NCs is the enhancement of the solid-state diffusion rates. This has made it possible to use nanoscale cation exchange and/or controlled interdiffusion as post-synthetic strategies to tailor the properties of NCs and hetero-NCs while preserving their size, shape, and heterostructure, by tuning their composition and/or elemental distribution profile [82, 104–122]. These techniques have also been recently used to achieve doping of semiconductor NCs [96, 97, 100].

The larger surface-to-volume ratio of NCs affects the optoelectronic properties. The best-known effect is that unshared atomic orbitals of surface atoms can give rise to localized energy levels within the HOMO–LUMO gap of the NC, which are known as trap states. These states can be detrimental to the PL quantum yield of the NC, if carrier localization into these states is followed by nonradiative exciton relaxation (i.e., energy dissipation as heat by coupling to vibrations) [16]. Radiative recombination between delocalized and trapped carriers may also occur, giving rise to PL that is strongly red-shifted with respect to the band-gap of the NC (the so-called trap PL). This emission is typically characterized by very broad bandwidths and low quantum efficiencies. It is thus often desirable to eliminate dangling bonds and defects at the surface of semiconductor NCs. This can be achieved by overcoating the NC either with a shell of a different semiconductor (thus forming a hetero-NC, see Sect. 2.2 above) or with suitable ligands that form strong bonds with the surface atoms, thereby shifting the energies of the surface states away from the HOMO–LUMO gap of the NC [16, 123]. Other ligands may in fact generate localized interfacial states or mid-gap states that trap one of the carriers and induce PL quenching (e.g., hole trapping by alkanethiols on CdSe QDs [82, 123, 124]), or directly shift the NC electronic states due to electrostatic or orbital mixing effects [64, 123, 125–127]. The capping ligand shell can be viewed as a self-assembled monolayer (SAM) at the surface of the NC [16, 123]. The internal structure of this SAM can also affect the PL of the NCs, either positively, by fostering surface reconstruction that eliminates trap states [16], or negatively, by imposing disorder to the surface [16, 128].

### Collective Effects in NC Superstructures: When 1 + 1 is Larger Than 2

An attractive feature of colloidal semiconductor NCs and hetero-NCs is that they can be used as solution-processable building blocks for nanostructured thin-films, either by directly depositing the colloidal suspension of NCs or hetero-NCs (the so-called NC inks) onto a substrate and evaporating the solvent [20, 23], or by allowing the NCs or hetero-NCs to self-organize into long-range three- or two-dimensionally ordered superlattices at air–liquid interfaces and subsequently transferring the superstructure to a suitable substrate [129, 130]. Colloidal NCs can also self-assemble into three-dimensionally ordered colloidal superparticles [131]. The geometry and properties of these superstructures can be tailored by the size, shape, composition and surface chemistry of the NC or hetero-NC building blocks [129–138]. In particular, surface ligands have been shown to have a dramatic impact on the directionality of the self-organization process [135, 139–143], leading in some cases to atomically aligned NC superlattices [135, 139, 143]. NC thin-films and superlattices hold promise for a variety of optoelectronic devices, such as light emitting devices, solar cells, photodetectors, and field-effect transistors [129, 130], since they may give rise to a number of novel properties dominated by collective interactions such as energy transfer, charge carrier transfer and migration, and inter-NC electronic coupling.

Another interesting type of superstructure is obtained by attaching colloidal semiconductor NCs (typically CdSe, CdTe, PbSe, or CuInS_2_) to nanostructured mesoporous films of wide band gap oxide semiconductors, such as TiO_2_ or SnO_2_. Depending on the band alignments, fast electron injection from the NC into the mesoporous film will occur, making it possible to use such superstructures as QD-sensitized solar cells, akin to the well-known dye-sensitized Grätzel solar cells [144–147].

## Excited-State Dynamics in Semiconductor Nanocrystals

In this section, we discuss the excited-state dynamics of semiconductor NCs and hetero-NCs, i.e., the processes that occur in a NC after excitation eventually leading to the emission of light. Following excitation, a NC makes the transition from one level to another until eventually relaxing back to the ground state. This sequence of events involves time scales that span over 15 orders of magnitude, from a few femtoseconds to a few seconds after photoexcitation. The possible relaxation pathways and the balance between their rates determine how efficiently light is emitted and at what wavelength. We will limit our discussion to processes occurring at room temperature, since these are more relevant for potential applications, and will thus neglect exciton fine-structure effects (see Sect. 2.1 above). Excited-state dynamics that are dictated by inter-NC interactions, such as energy migration [35, 130, 148–150] and charge carrier transport [130, 151], or energy transfer between NCs and acceptor molecules (usually referred to as Förster resonance energy transfer, FRET) [31, 35, 152] are also beyond the scope of this review, and we refer the interested reader to prior publications that focus on these aspects.

### Relaxation of Hot-Carrier States: fs to ps Timescales

**Fig. 4:**
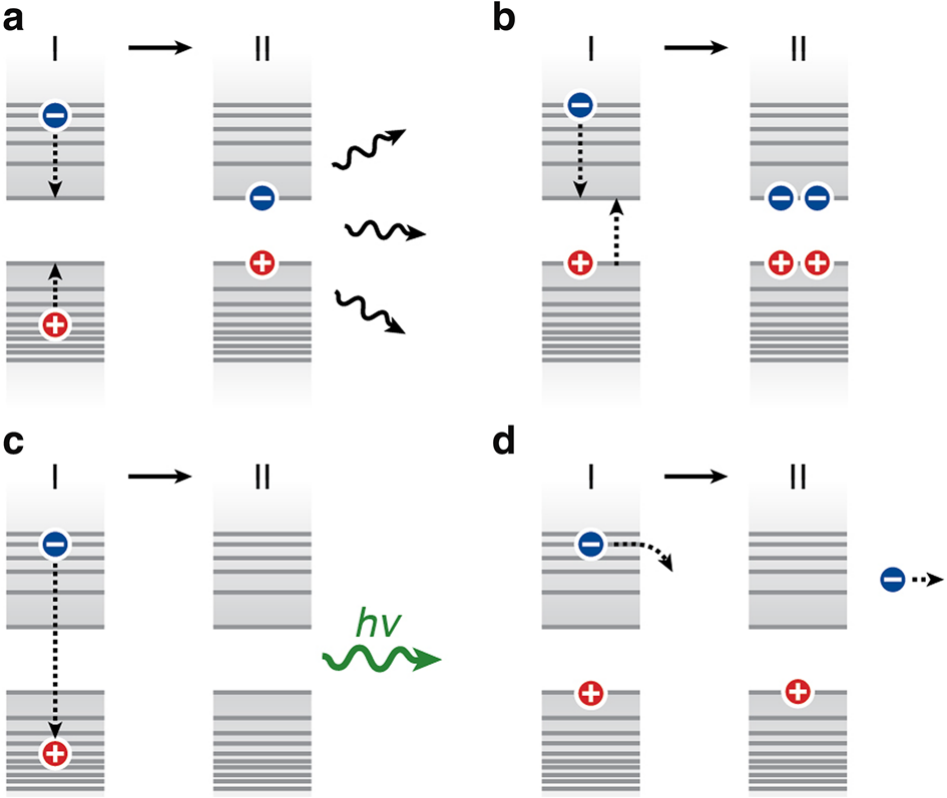
Schematic representation of possible relaxation pathways for hot-carrier states. **a** Thermalization by means of electron–hole Auger coupling and/or coupling to vibrations. **b** Multi-exciton generation, where the hot-carrier energy is converted into an additional electron–hole pair. **c** Hot-exciton emission. **d** Ejection of a hot charge carrier to the environment of the NC

Directly after photoexcitation a NC is in a high-energy state, where usually both electron and hole occupy levels deep in the conduction and valence band. In other words, they have energy in excess of the band edge, and are usually referred to as “hot carriers”. Typically the excess carrier energy is rapidly lost as heat on a picosecond timescale or faster [153–161] (Fig. 4a). Studies of this cooling process, and attempts to suppress it, have until now mainly focused on Cd- and Pb-based NCs, using photoluminescence spectroscopy and transient absorption spectroscopy. The mechanism of rapid cooling is not precisely known, but thought to involve coupling to vibrations as well as Auger-coupling between electrons and holes [51, 162–165].

**Fig. 5:**
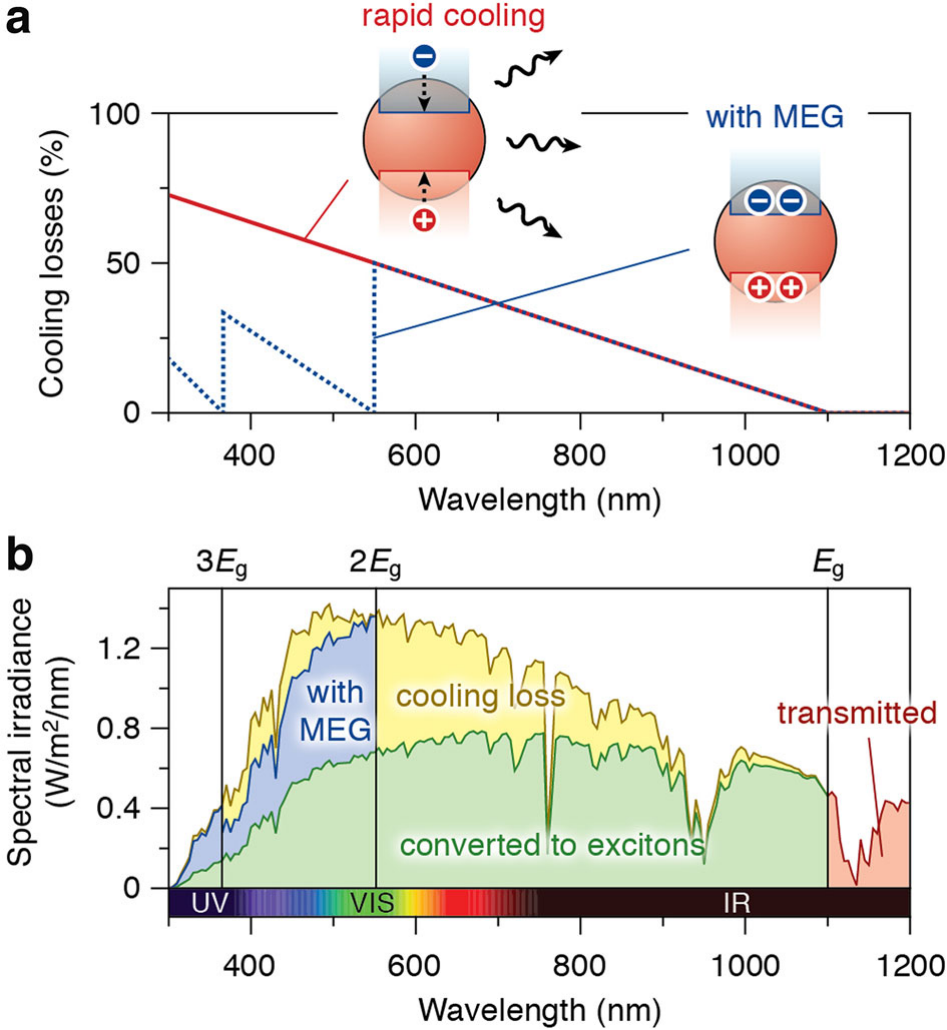
Multi-exciton generation for solar cell applications. We consider a QD solar cell with a band gap of 1.1 eV (=1100 nm). **a** The fraction of energy lost due to cooling in the transition from light to excitons in a QD. The* red line* denotes the situation where all excess carrier energy is lost by cooling. For example, absorption of a photon with an energy of 2 eV (=620 nm; *red*) results in an electron–hole pair with an energy of 1.1 eV (=the bandgap), while 0.9 eV is lost by cooling. The* blue dashed line* is the optimal situation where all excess carrier energy is used for multi-exciton generation. For example, a photon of 2.2 eV (=565 nm; *yellow/green*) has sufficient energy to create two electron–hole pairs with a combined energy of 2.2 eV, and no cooling losses. **b** The solar spectrum (AM1.5), with the potential benefit of multi-exciton generation indicated. Without multi-exciton generation, only the* green shaded area* is converted to excitons, while the* yellow* and* blue* (30 % of the total solar intensity) are lost to cooling. Optimal multi-exciton generation can prevent cooling losses of the* blue shaded area* (34 % of the total cooling loss). The wavelengths corresponding to once, twice, and three times the QD band gap are indicated, as well as the part of the solar spectrum that does not match the QD absorption (*red shaded area*)

There has been interest in making use of hot-carrier energy in NCs, by reducing the cooling efficiency. The possibility of multi-exciton generation (MEG), also called carrier multiplication (CM), has been investigated for several years, most commonly in Pb-chalcogenide NCs [166–172], but also for other NC materials [173–176]. In the process of multi-exciton generation, a hot carrier with excess energy higher than the bandgap can relax to the ground state while generating an additional electron–hole pair (Fig. 4b). This process has been theoretically predicted to happen on a fs to ns timescale [177]. Multi-exciton generation has the potential to increase the efficiency of QD solar cells to above the Shockley-Queisser limit [178], and is therefore of great interest (see Fig. 5). Many studies have reported the possibility of efficient MEG in NCs, but other studies have challenged too optimistic values for efficiency and energy threshold [169, 175, 176]. As a variation to multi-exciton generation in *individual* QDs, the phenomenon of space-separated quantum cutting has been reported for *ensembles* of QDs of Si. Here, the hot-carrier energy in one QD is transferred to a neighboring QD, after which both can emit [179, 180].

NCs can show direct light emission from hot-exciton states (Fig. 4c). *Interband* hot-carrier emission is due to recombination of a hot carrier in one band (e.g., an electron in the conduction band), with a carrier in the other band. This emission is blue-shifted with respect to that from the ground-state exciton, and decays on a timescale of picoseconds or faster [73, 172, 181]. In addition, the possibility of *intraband* hot-carrier emission has recently been demonstrated in Cd-based and Hg-based NCs [182, 183]. In this process, a hot carrier relaxes to a lower energy level in the same band by the emission of an infrared photon. A particular variation of hot-carrier interband emission occurs in hetero-NCs, if charge carrier localization to the equilibrium situation (as according to the band alignment; see Fig. 3) is inhibited. For example, in CdSe/CdS hetero-NCs hole localization from the high-bandgap material CdS to the CdSe core can be suppressed at high excitation power when multiple mutually repulsive valence band holes simultaneously co-exist in the hetero-NC (Coulomb blockade effect). It has been shown that this leads to significant emission from the CdS arms in CdSe/CdS tetrapods [184–186] or from the CdS shell in CdSe/CdS dot-in-bulk NCs [187].

Another way to reduce the loss of hot-carrier energy is to offer charge transfer pathways that compete with cooling (Fig. 4d). To achieve this, a charge transfer time constant of at most a few picoseconds is necessary. Hot-electron transfer on femtosecond timescales has been demonstrated from PbSe QDs to TiO_2_ [188]. Moreover, transfer from hot-carrier states inside the QD to states on the surface or in the environment has been proposed to contribute to photo-ionization and blinking [189, 190]. Charge transfer from hot-electron states is of potential use for solar cell applications, where currently thermalization constitutes a major part of the energy conversion losses [191] (see also Fig. 5b). To enable efficient hot-electron transfer, the competing process of cooling must be suppressed. This could be achieved in designed hetero-NCs to decouple the hot electron from the hole in the valence band as well as from ligand vibrations [51].

### Auger Decay of Multi-Carrier States: ps to ns Time Scales

**Fig. 6:**
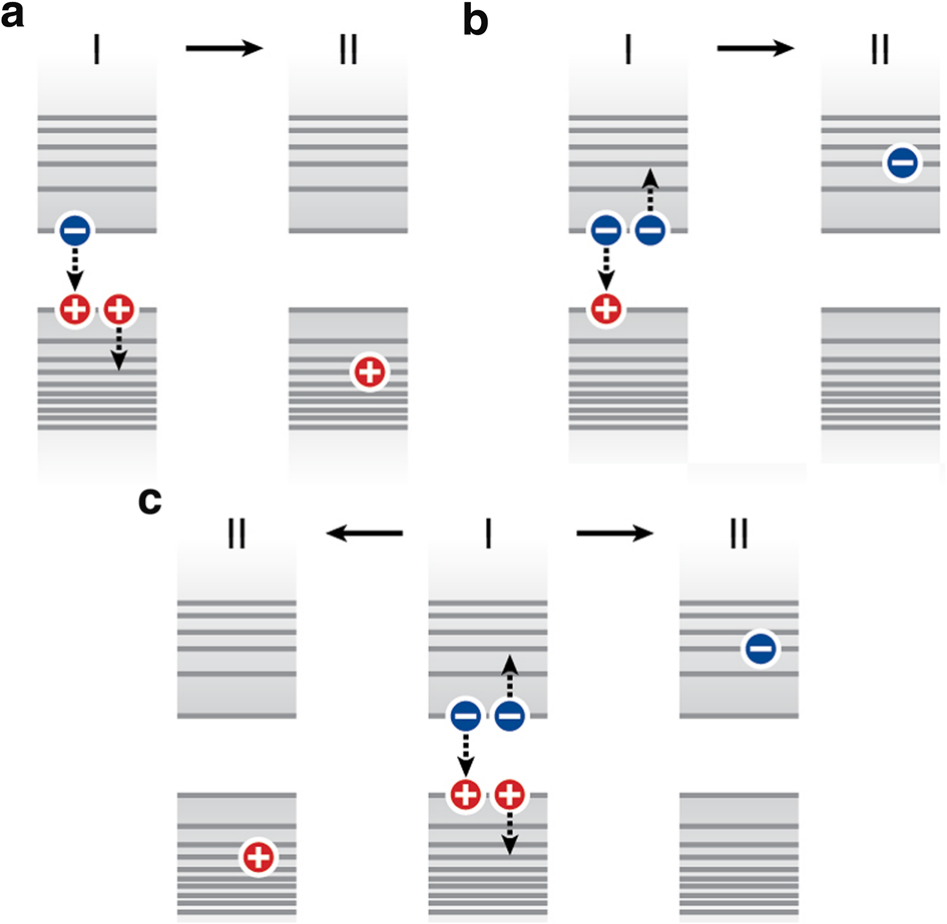
Examples of Auger processes in semiconductor NCs in the trion (i.e., charged) or biexciton state. **a** In the positive trion state, the recombination energy of an electron–hole pair can be transferred to the additional hole. **b** In the negative trion state, the recombination energy of an electron–hole pair can be transferred to the additional electron. **c** In the biexciton state, the recombination energy of an electron–hole pair can be transferred either to the additional hole (the “positive trion pathway”; to the *left*) or to the additional electron (the “negative trion pathway”; to the *right*) [201]

After the carriers have cooled down to the edges of valence and conduction band via the pathways depicted in Fig. 4, the next important relaxation pathway is Auger decay. An Auger process is the transfer of energy from one charge carrier in the NC to another. This process plays an important role in semiconductor NCs whenever there are three or more charge carriers present, of which at least one electron and one hole. The most common NC states likely to undergo an Auger process are trion states, i.e., charged states with an electron–hole pair in the NC as well as an additional charge carrier in a quantum confined energy level, and the biexciton state, i.e., the state with two electron–hole pairs in the NC. Figure 6 illustrates the most commonly considered Auger processes. These are Auger processes that involve charge carriers in delocalized levels (i.e., the quantum confined orbitals that extend over the entire NC). The final state of an Auger process always has a charge carrier in a highly excited level (situations II in Fig. 5), which then usually undergoes rapid cooling as in Fig. 4a. The net result of an Auger process is therefore energy loss as heat. Trapped charge carriers can be involved in Auger processes in NCs [192–194]. Auger processes involving trapped charge carriers are poorly understood and not further discussed here, but should be investigated further.

For the use of semiconductor NCs as photoluminescent centers, e.g., in LEDs, laser gain material, or biomedical tracers, it is usually desired that optical cycling is as efficient as possible. This means that every photon absorbed should lead to a photon emitted. Therefore, to minimize Auger losses, the NC must be uncharged (i.e., no trion Auger decay; Fig. 6a, b) and the excitation intensity must be sufficiently low to prevent the generation of biexcitons (Fig. 6c). However, under illumination NCs charge up intermittently and seemingly randomly, leading to a phenomenon known as PL intermittency or “blinking” (see Sect. 3.4 below) [195, 196], while for applications such as lasing high excitation intensities are a necessity [14, 197, 198]. As a result, the possibility of Auger quenching cannot always be avoided. It should be noted that at the highest operating powers in lasers, stimulated emission easily outcompetes Auger recombination, but Auger recombination nevertheless negatively affects the lasing threshold in typical QD lasers.

**Fig. 7:**
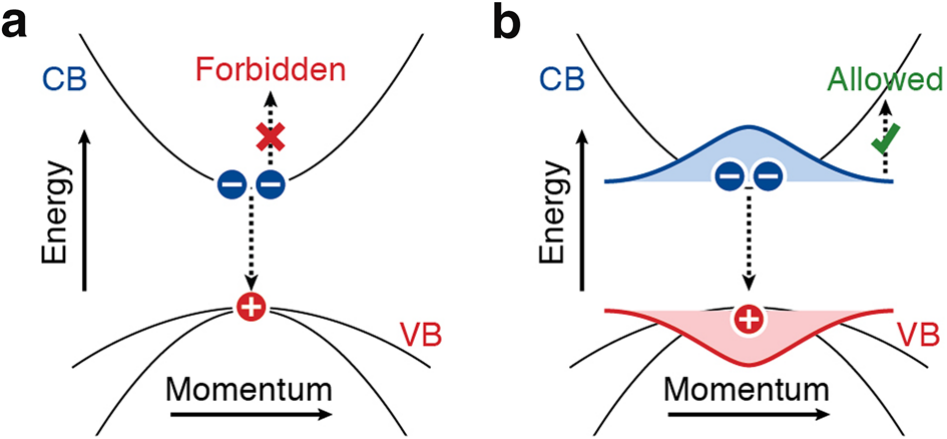
A simple picture of momentum conservation for Auger processes in NCs. Negative trion Auger recombination is depicted in the electronic dispersion diagram of a typical direct-gap semiconductor. **a** Recombination of an electron–hole pair at the band edge of a bulk semiconductor involves no change in translational momentum (*vertical downward arrows*). The excess electron can therefore not accept the recombination energy to make an energy and momentum conserving transition. **b** In a NC, on the other hand, the quantum confined energy levels have no well-defined translational momentum. In other words, the charge carrier wavefunctions contain many spatial frequency components (*red* and *blue shaded* *areas*). The momentum selection rule is therefore not so strict in a NC. Consequently, the Auger process can be much faster, depending on the overlap between charge carrier wavefunctions in momentum space [224, 225]

In recent years, considerable research efforts have been devoted to understanding Auger decay in semiconductor NCs [199–201]. The timescale of Auger processes is typically on the order of 1–100 ps in single component NCs such as PbSe [199], Si [173], PbS [202], CdTe [203], Ge [204], InAs [205], or Pb-perovskites [161]. The Auger timescales in NCs are faster than those in the corresponding bulk material [204]. The difference in Auger rates between bulk (slower) and nanocrystalline (faster) materials, is believed to be due to two reasons. First, all interactions between charge carriers in NCs, including Auger interaction, are enhanced because of their spatial and dielectric confinement (i.e., they are spatially confined in a small volume with high dielectric constant *ε*_1_, which is embedded in a medium with lower dielectric constant *ε*_2_). This increases the Coulomb interaction energy between carriers, which mediates Auger scattering. Second, the conservation rule for translational momentum that suppresses Auger processes in bulk materials is less strict in NCs, because spatial confinement leads to uncertainty in momentum (Heisenberg principle). Figure 7 schematically illustrates the momentum selection rule that governs Auger interaction rates in NCs, using negative trion decay as an example.

The trion Auger dynamics in semiconductor NCs have been investigated under pulsed excitation using photoluminescence measurements. Under strong excitation, NCs charge intermittently (see also Sect. 3.4), which allows one to investigate the properties of trions on the single-NC level [72, 206–208]. However, this method is only applicable to NCs where the trion state luminescence can be clearly distinguished from the neutral exciton luminescence. For many types of NCs, the blinking behavior is complicated by the involvement of multiple states [209], and one cannot rely on random charging to investigate charged states. Methods of controlled electrochemical or photochemical charging of luminescent NCs have been developed as an alternative for the studies of luminescence from charged NCs [190, 210, 211]. Not only do these methods offer control over the charge state of QDs, they also allow for statistically significant measurements on entire ensembles [211], while single-NC experiments are necessarily limited to a small number of NCs.

Ensemble transient absorption experiments are most commonly used to study multi-exciton dynamics in NCs [161, 173, 199, 203–205]. Analysis of the fast photoluminescence decay components of NCs under strong excitation can also provide information about multi-exciton decay rates [170, 212]. Both methods rely on a significant multi-exciton population in the NCs, and therefore require strong laser excitation. One must be careful that under such conditions, the interpretation of data can become complicated if NCs charge up or defects are generated [157, 161]. Alternatively, information about biexciton dynamics and quantum efficiencies can be obtained from photon correlation analysis on the single-NC level [213, 214] or the ensemble level [215].

Auger recombination negatively affects the performance of NCs for applications such as light emitting diodes [216], lasers [84], or solar cells under concentrated illumination [217]. As discussed above, Auger processes in NCs are rapid and efficient because of spatial confinement of charge carriers. Therefore, the most obvious way to reduce Auger recombination rates is to increase the size of NCs [199, 201, 204, 211, 218]. However, this may not always be a desired strategy if one wants to make use of quantum confinement effects to tune the electronic properties of NCs. For more subtle control over Auger processes, hetero-NCs have been developed with designed charge carrier confinement potentials [219, 220]. The most commonly studied hetero-NC composition for reduced Auger losses is CdSe/CdS in various shapes and sizes [72, 84, 206, 221–223]. It was first predicted theoretically [224, 225] and later confirmed experimentally [84, 221–223] that an alloyed core–shell interface leads to suppressed Auger recombination. An alloyed hetero-interface creates a smooth confinement potential for charge carriers in which high-momentum components in the wavefunctions are reduced in amplitude (see Fig. 7). Indeed, Auger recombination rates in NCs are strongly dependent on the exact size and shape of the NC [201], leading to wide variations in Auger dynamics within a NC ensemble [226, 227]. Hence, it seems that the careful design of uniform ensembles of hetero-NCs with smooth confinement potentials is the pathway to NCs with reduced Auger losses.

### Radiative Decay in Semiconductor Nanocrystals: ns to µs Time Scales

The most studied electron–hole recombination channel in semiconductor NCs is spontaneous radiative decay. Not only do many experimental methods rely on the detection of photons emitted in a radiative decay pathway, but radiative decay of excited NC states is also often the desired pathway for applications. Several types of spontaneous radiative decay are possible in semiconductor NCs, depending on the composition. For most NC compositions under moderate excitation, radiative decay is predominantly due to recombination of two delocalized charge carriers (electron and hole) in the lowest-energy quantum confined states of the two respective bands. The electron and hole wavefunctions, and hence the characteristics of the emission, are determined by the composition, size and geometry of the NC, as illustrated in Figs. 1, 2 and 3. With the development of NCs and hetero-NCs of a wide variety of sizes, shapes, and compositions, precise control over spontaneous emission from the lowest-energy exciton has been achieved. Radiative recombination of a delocalized carrier with a trapped (i.e., localized) carrier may also occur (trap PL), but is usually characterized by low quantum efficiencies, since carrier trapping favors non-radiative decay pathways by decreasing the electron–hole wave function overlap while increasing the coupling between the localized carrier and its immediate vicinity. In the case of NCs doped with luminescent ions (e.g., ZnSe:Mn^2+^ [228] or LaPO_4_:Tb^3+^ [229]), radiative recombination occurs primarily at the dopant.

In this section, we discuss what determines the photoluminescence quantum efficiency of NCs and the rate of radiative decay. Auger quenching diminishes the quantum efficiency of the emission when a NC is charged or when multiple electron–hole pairs are present (see Sect. 3.2 above). However, uncharged NCs under weak illumination may find alternative non-radiative decay pathways, which lower the quantum efficiency. Below, spontaneous radiative decay in semiconductor NCs is first discussed, followed by non-radiative decay pathways that can lead to a photoluminescence quantum efficiency below unity.

The rate of radiative decay of a delocalized electron–hole pair in a semiconductor NC can be estimated as [36, 230]

1 $$\gamma_{\text{rad}} = C\lambda^{ - 1} \rho K .$$

Here *C* is a pre-factor that depends on the type of semiconductor, *λ* is the emission wavelength, and *ρ* is the density of optical states experienced by the exciton (see below for further explanation). *K* is the electron–hole overlap integral squared.

**Fig. 8:**
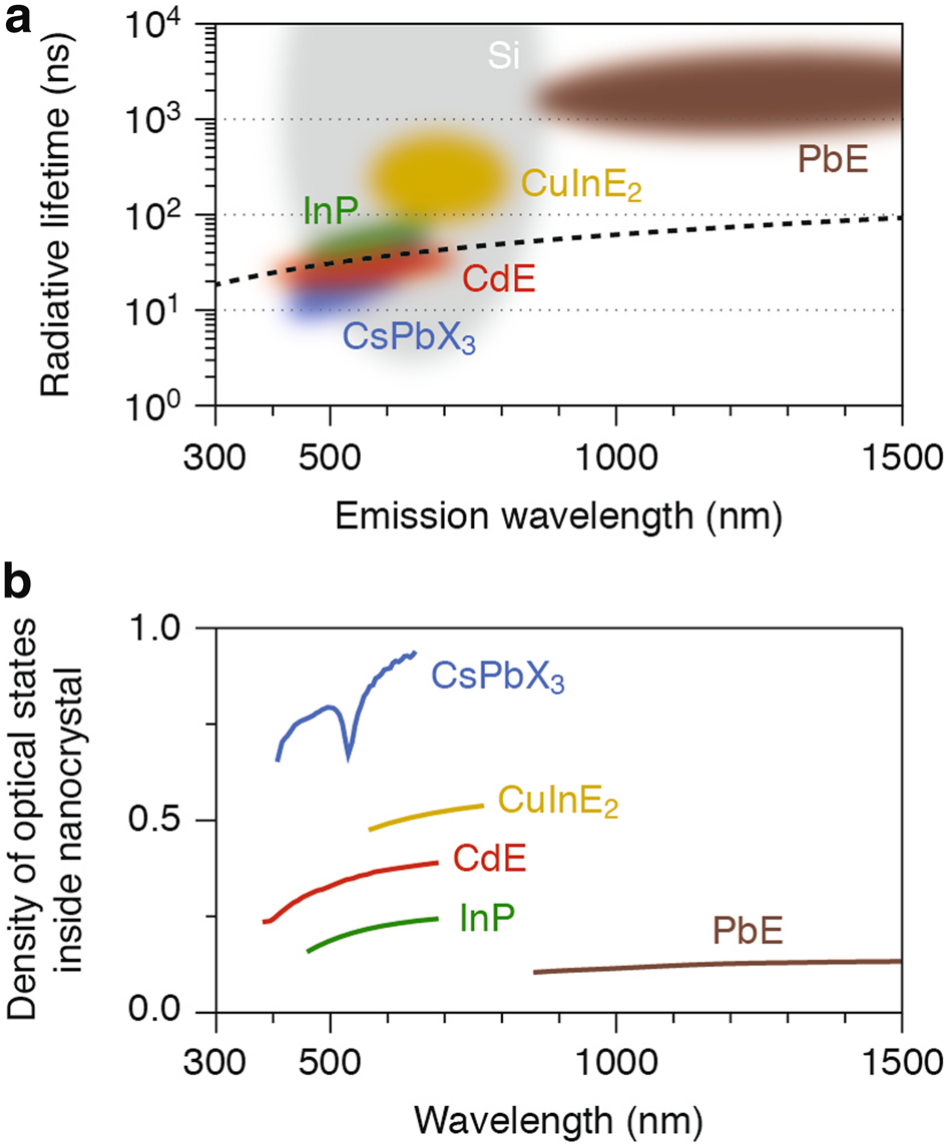
The rate of radiative decay in colloidal quantum dots. **a** Typical radiative lifetimes and emission wavelengths obtainable for some common and emerging QDs: lead chalcogenides (PbE with E = S, Se; *brown*) [231, 232], copper indium chalcogenides (CuInE_2_ with E = S, Se; *yellow*) [233, 234], cadmium chalcogenides (CdE with E = Se, Te; *red*) [58, 235], indium phosphide (InP; *green*) [48], silicon (Si; *gray*) [236], and cesium lead halides (CsPbX_3_ with X = Cl, Br, I; *blue*) [161, 237]. The differently colored clouds indicate the approximate range of combinations for radiative lifetime and emission wavelength that can be found in these materials. The* dashed line* denotes a linear dependence between radiative lifetime and emission wavelength (see text for details). **b** The density of optical states *ρ* (Eq. 2) for QDs of various semiconductor materials dispersed in a medium with refractive index *n* = 1.5, such as toluene or poly(methyl methacrylate), normalized to the density of optical states in vacuum. The refractive index data were taken from Ref. [306] for CsPbBr_3_ (*blue line*), from http://www.filmetrics.com for CdTe (*red*), InP (*green*), and PbS (*brown*), and from Ref. [307] for CuGaS_2_ (*yellow*; as a close analogue of CuInE_2_ materials)

Figure 8a shows typical values for the radiative lifetime (the inverse of the radiative decay rate) in common and emerging types of zero-dimensional NCs (i.e., QDs): lead chalcogenides (PbE; E = S, Se) [231, 232], copper indium chalcogenides [233, 234] (CuInE_2_; E = S, Se), cadmium chalcogenides (CdE, E = Se, Te) [58, 235], indium phosphide (InP) [48], silicon (Si) [236], and cesium lead halides (CsPbX_3_) [161, 237]. A rough trend is visible that radiative lifetimes are longer for longer emission wavelengths. Indeed, Eq. (1) shows that, for a given QD material, the radiative lifetime (which is the inverse of $\gamma_{\text{rad}}$) should scale linearly with emission wavelength. Nevertheless, the various QD materials deviate from a general linear dependence between radiative lifetime and emission wavelength (dashed line). These deviations are due to variations in the pre-factor *C* and the density of optical states *ρ*.

The pre-factor *C* is a material-specific constant that accounts for the electronic properties of the QD material. In PbE, CdE, InP, and CsPbX_3_, where the radiative decay is due to recombination of delocalized electron–hole pair (i.e., the lowest energy exciton), *C* depends for example on how strongly light couples valence and conduction band states, and also the exciton fine-structure [36].

The density of optical states *ρ* is a factor of potentially large influence on the radiative decay. It depends on the refractive index of the QD, the shape of the QD, and the polarization of the emission, as well as on the photonic environment of the QD. For example, photonic crystals [238] or plasmonic structures [239] can enhance or suppress radiative decay of QDs. For QDs dispersed in an organic medium, as they are commonly prepared and analyzed, the density of optical states *ρ* is determined by the refractive index of the medium *n* and the refractive index contrast with the QD material itself *n*_QD_:

2 $$\rho = n\left| {\frac{{3n^{2} }}{{2n^{2} + n_{\text{QD}}^{2} }}} \right|^{2} .$$

Indeed, the radiative decay rate of excitons in core–shell QDs [240] and of luminescent doped ions in NCs [226] depends on the solvent refractive index as described by Eq. (2). The factor $\left| {\frac{{3n^{2} }}{{2n^{2} + n_{\text{QD}}^{2} }}} \right|^{2}$ in Eq. (2) is also known as the local-field factor, and describes the effect of the refractive index contrast between the QD and the surrounding medium. Figure 8b shows that the values of the local-field factor range over one order of magnitude for different QD materials.

The electron–hole overlap integral squared [230] can be expressed as

3 $$K = \left| {\mathop \int \nolimits \psi_{\text{e}} \left( {\mathbf{r}} \right)\psi_{\text{h}} \left( {\mathbf{r}} \right){\text{d}}{\mathbf{r}}} \right|^{2}$$

where $\psi_{\text{e}} \left( {\mathbf{r}} \right)$ and $\psi_{\text{h}} \left( {\mathbf{r}} \right)$ are the electron and hole wavefunctions. The factor *K* is commonly used to reduce the radiative decay rate of excitons in NCs, by making type-I½ or type-II hetero-NCs in which the electron and hole are spatially separated [241] (see Sect. 2.2; Fig. 3 above). Reduced rates of spontaneous radiative decay can be useful for applications such as in lasers or photodetectors, where spontaneous emission is not the desired decay pathway for charge carriers. The mechanism of radiative recombination in some QD materials such as Si or CuInE_2_ is believed to involve at least one localized charge carrier [24, 32, 236, 242], and therefore cannot be expected to follow the trend predicted by Eq. (1). It is not yet clear how hetero-NCs with engineered electron and hole wavefunctions can be used to control radiative decay rates in QDs of materials such as Si or CuInE_2_ [117].

The quantum efficiency *η* of NC emission is determined by the competition between the radiative decay rate *γ*_rad_ and all the possible non-radiative decay pathways with a combined rate *γ*_nr_:

4 $$\eta = \frac{{\gamma_{\text{rad}} }}{{\gamma_{\text{rad}} + \gamma_{\text{nr}} }}$$

As discussed in Sect. 2.3 above, imperfections in the NC such as crystal defects or unsaturated chemical bonds on the surface have been identified as an important factor determining the quantum efficiency [123]. They provide “trap states” for charge carriers, i.e., energy levels within the bandgap where the charge carrier is spatially localized. Indeed, the quantum efficiency of NC emission improves when the NC surface is covered with a protective shell of high-bandgap material [13, 15, 16, 61, 74, 243–246], or when ligands saturate chemical bonds on the surface [16, 123, 247–251].

**Fig. 9:**
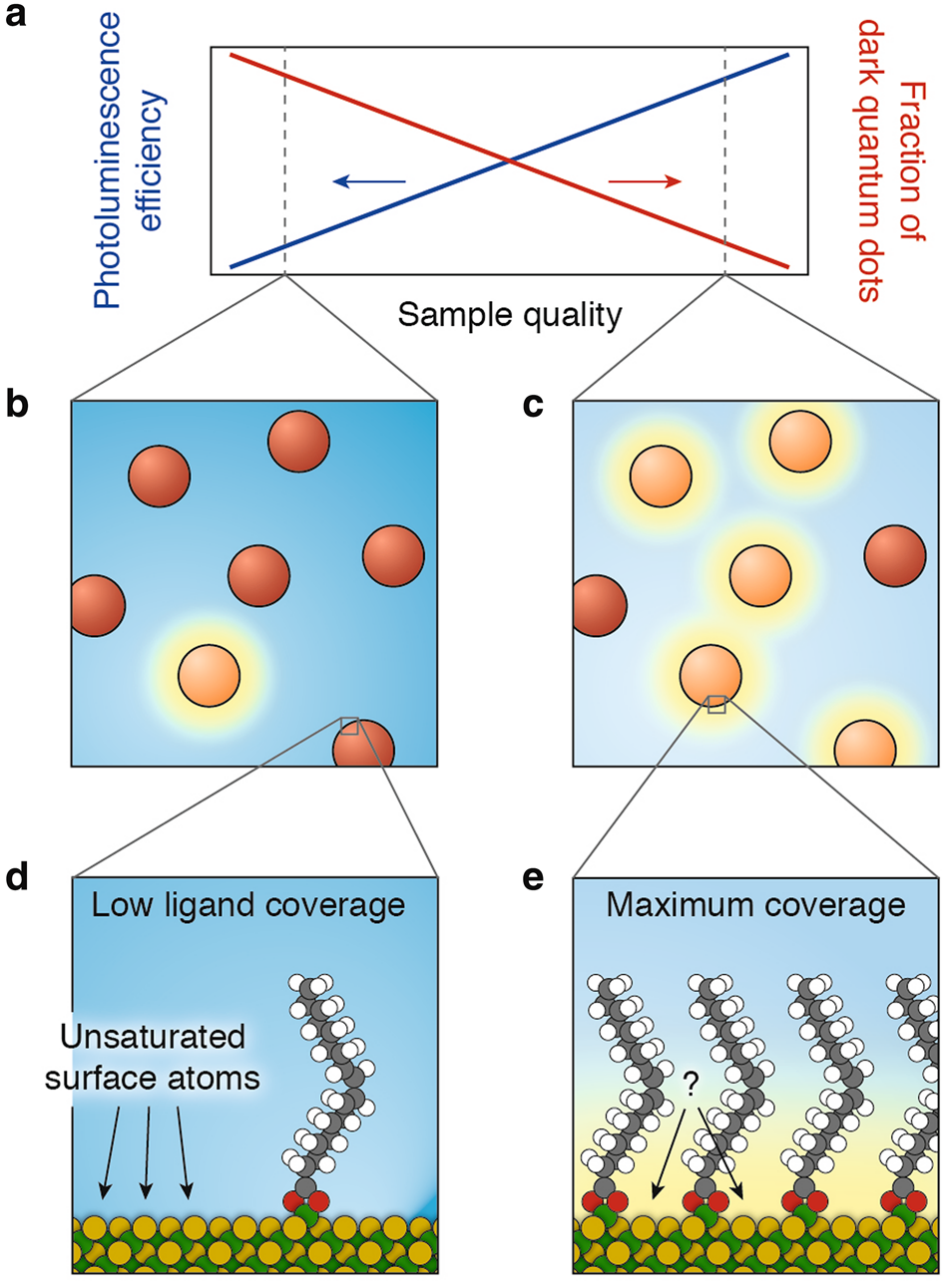
The fraction of dark quantum dots in a sample. **a** With increasing quality of a QD sample, the photoluminescence efficiency improves (*blue line*). This happens not only because the brightness of each individual QD increases, but mainly because the number of completely dark QDs in the sample decreases (*red line*). **b** A low-quality sample contains many dark QDs and only a few bright ones. **c** A high-quality sample contains fewer dark QDs. **d** The surface of a dark CdSe QD: Cd (100) surface with a single Cd(oleate)_2_ ligand attached, leaving many unsaturated surface atoms, which are believed to act as charge carrier traps enabling non-radiative recombination. **e** The sample quality improves when the QD surfaces are covered by ligands [251]. However, the maximum ligand coverage set by steric hindrance (~3 nm^−2^ for oleic acid) is not sufficient to saturate all surface atoms (~6 nm^−2^ for CdSe {100} or CdSe {111}) [123]. There are still unsaturated surface atoms, as highlighted with a* question mark*. Panels **d** and **e** were adapted from Ref. [123]

Generally, the quantum efficiency of different NCs within a single synthesis batch varies strongly. Some NCs have a high quantum efficiency (near 100 %), while others have a quantum efficiency near 0 %. The two subpopulations in a NC ensemble are also known as the “bright fraction” and the “dark fraction” [251–256] (see Fig. 9). This means that within a single batch, some NCs have (almost) no non-radiative decay pathways, while in other NCs non-radiative decay is very likely. The dark fraction hardly contributes to the observed photoluminescence and, as a result, the photoluminescence decay measurements will reflect the dynamics of the bright NCs. This makes it possible that the photoluminescence decay curve for NC samples with ensemble quantum efficiencies well below 100 % are nearly single-exponential, with a time constant equal to the radiative lifetime of the NCs. High-quality NC batches are thus brighter not necessarily because non-radiative decay is suppressed in each *individual* NC, but rather because the fraction of completely dark NCs in the ensemble is smaller. This is schematically depicted in Fig. 9a–c.

Despite over 20 years of research, the mechanisms of photoluminescence quenching by charge carrier trapping are still poorly understood. The simplest picture is that quenching can be suppressed by saturating chemical bonds of the surface atoms [15, 16, 61, 243–249, 251]. This can be achieved by overcoating the NC either by a shell of another semiconductor or by a ligand layer. For example, the photoluminescence quantum efficiencies of CdSe QDs can be increased to values as high as 85 % either by overcoating with CdS shells or by capping with primary alkylamines such as hexadecylamine. Computational studies have shown that linear chain alkylamines can form densely packed monolayers at the surface of CdSe NCs, saturating all the available surface Cd atoms [257]. Nevertheless, bright NCs can exist in a sample with low photoluminescence quantum efficiencies, low average surface quality and low average surface coverage by ligands (Fig. 9b, d). Many commonly used surface ligands, such as oleic acid, are bulky and can only saturate half of the available surface atoms because of steric hindrance [123]. This means that even the brightest NCs in an ensemble of oleic acid capped NCs have imperfect saturation of surface atoms. Clearly, additional factors affect the electronic structure of the NC surface, and charge carrier trapping. For example, trap states can be eliminated by surface relaxation and/or reconstruction in such a way that the dangling orbitals of neighboring cations and anions partially overlap, leading to a redistribution of electronic density that makes the surface auto-compensated (a process known as self-passivation or “self-healing”) [258]. Surface- and global reconstruction has been observed for NCs of several compositions (e.g., CdSe, ZnSe) [259, 260], and shown to be affected by the nature and structure of the capping ligand monolayer [128, 261]. Nevertheless, it is currently unknown how the atomic structures, including surface ligands, of bright and dark NCs differ [262]. Some recent successes in correlated optical and electron microscopy have been reported, that can lead to more insight into the microscopic nature of quenching [222, 263, 264]. For example, a lower (time-average) brightness of NCs has been connected to stacking faults in the crystal structure or imperfections in surface coverage by a high-bandgap semiconductor shell [263]. The very dynamic nature of the NC surface and the strong interplay between capping ligands and the inorganic core [16] are also likely important factors determining charge carrier trapping. Unfortunately, the organic surface ligands are invisible in electron microscopy and can currently be investigated only on the ensemble level using infrared absorption [249, 265, 266], nuclear magnetic resonance spectroscopy [249, 267, 268], or neutron scattering [269, 270].

### Blinking Dynamics on ms Timescales and Slower

Interestingly, the timescales relevant to the optical properties of semiconductor NCs extend to much longer than the radiative lifetime of the exciton. In this section, we do not discuss irreversible bleaching of NC luminescence due to, for example, oxidation [271], but only reversible physical phenomena encountered in NCs on timescales beyond the exciton radiative lifetime. These include not only photoluminescence intermittency (blinking), but also photodimming and photobrightening, spectral diffusion, and delayed emission. We will first give a brief overview of experimental studies, and then discuss the microscopic nature of blinking and related processes, which is still largely unknown.

**Fig. 10:**
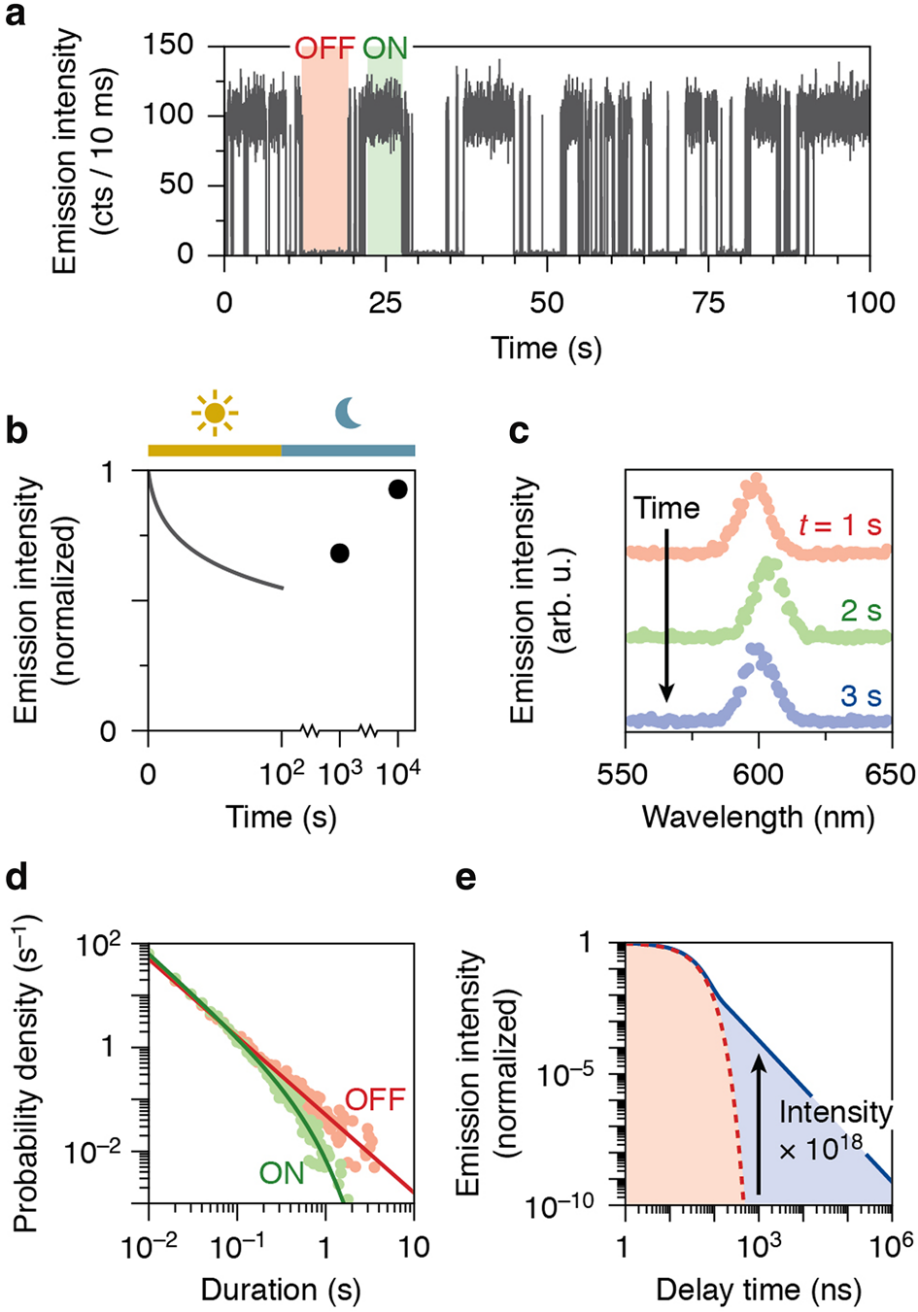
Slow dynamics in semiconductor nanocrystals. **a** Under continued excitation, the emission from a typical individual NC turns on and off intermittently on time scales of milliseconds to many seconds. **b** If the durations of on and off periods have different distributions (see panel **d** for typical statistics), then the brightness of an ensemble of NCs can decrease under continued illumination as more NCs enter an off state. The brightness is restored in the dark. **c** The emission spectrum of an individual NC shifts back and forth over a few nanometers on time scales of seconds. **d** The distribution of on (*green*) and off (*red*) durations typically shows power-law statistics with exponents of approximately 1.5. The on statistics exhibit an exponential cut-off that depends on the excitation power. **e** The photoluminescence decay following pulsed laser excitation of NCs shows an exponential component due to radiative recombination of an electron–hole pair (*red*; see Sect. 3.3). On time scales longer than a few nanoseconds, the emission is dominated by a power-law component of delayed emission due to reversible charge carrier trapping (*blue*). All data in this figure are simulated

In 1996, Nirmal et al. [195] observed that the luminescence from NCs turns on and off intermittently on time scales from milliseconds up to many seconds (see Fig. 10a). This phenomenon, commonly known as “blinking”, becomes apparent in studies on individual NCs, but is hidden in ensemble measurements on many NCs simultaneously. Nevertheless, blinking does have an adverse effect on the properties of NC ensembles, because there is always a fraction of NCs in the non-emissive state. Interestingly, because of the peculiar statistics of blinking, the fraction of non-emissive NCs can change in time under continued excitation, leading to reversible photodimming or photobrightening over time scales of many seconds (Fig. 10b) [272–274].

Blinking is observed in many different types of NCs, including QDs of CdSe [195], InP [275], CdTe [276], PbS [277], InAs [278], Pb-perovskites [279], as well as various hetero-NCs, and even in very different emitters such as organic dye molecules [280]. Methods to reduce blinking of NCs (i.e., to make random switches to a dark state less frequent) include surface protection using organic ligands [281] or an inorganic shell material [195, 282, 283], and also plasmonic enhancement of radiative decay [284]. This indicates that blinking involves slow changes on the surface of the QD that introduce non-radiative decay pathways. These can be either geometrical changes induced by ligand adsorption and desorption, or charge carrier trapping (for more discussion, see below).

A second phenomenon fundamental to semiconductor NCs but only observable in single-emitter measurements, is spectral diffusion (Fig. 10c). This entails that over time the emission spectrum of a NC shifts or jumps back and forth over the range of a few nanometers. Temporal variations in the peak emission wavelength are accompanied by, and correlated with, variations in the emission line width [285–288]. Most of the experiments into spectral diffusion were conducted at cryogenic temperatures [289–292], but the process occurs at room temperature, too [285–288]. A direct link between spectral diffusion and blinking was proposed, based on correlations between blinking events (on–off switches) and spectral shifts [290, 293]. At room temperature, typical time scales for spectral diffusion are milliseconds to seconds, but not shorter [294].

The statistics of blinking are peculiar. Typical duration distributions of bright and dark periods in an emission trace (as in Fig. 10a) are depicted in Fig. 10d. The durations are power-law distributed (with an exponential cut-off for the bright periods at long time scales) [295]. This means that a bright or dark period is most likely short (only a few milliseconds), but much longer periods of many seconds occur as well. The range of durations is much wider than it would be in case of exponential statistics. The power-law exponents are around 1.5 for most NCs, and independent or nearly independent of temperature [276], excitation intensity [276], and nature of the excitation laser (continuous wave or pulsed) [296]. Interestingly, the band-edge emission of semiconductor NCs contains a slow “delayed” component with power-law statistics, that extends over time scales from nanoseconds up to (at least) milliseconds [240, 256, 297] (Fig. 10e). This very slow emission is not trap emission due to recombination of a trapped and a delocalized charge carrier (see Sect. 2.3 above), because the emission wavelength is (nearly) identical to the band-edge emission. Instead, this emission component has been ascribed to reversible charge carrier trapping and detrapping, followed by emission [298]. Based on the similar statistics, a close relation between blinking and delayed emission has been suggested [240, 297].

The microscopic nature of blinking is, after 20 years, still under debate. Models must contain at least two ingredients: they must explain what makes a NC dark in the off state, and what causes the characteristic power-law statistics. Early on, Efros and Rosen [196] proposed the charging–discharging model, where the NC can become charged by the ejection of a photogenerated charge carrier. A neutral NC would correspond to the on state, while a charged NC is in the off state, where photoluminescence is quenched by Auger recombination (see Sect. 3.2). The initial model [196] proposed that charging could be due to Auger ejection of a charge carrier following the generation of a biexciton, but this would result in exponential statistics. Several adaptations for the charging–discharging model have been developed to account for the power-law statistics of blinking. These adapted models assume that the rate of charging and/or discharging varies in time, because of Coulomb blockade [299] or fluctuations in the geometry and surface structure of the NC [300], or because tunneling barriers for charge carrier trapping vary slowly in height and width [295].

The picture of Auger quenching in the off state has later been challenged, based on comparisons between the quenching rate of the biexciton state (due to Auger processes) and the off state [301]. However, such comparison assumes that Auger quenching in the off state is only due to the remaining charge carrier in the NC, while the ejected charge carrier does not play a role. Taking this role into account [193], may explain the discrepancies between biexciton and off state quenching. As an alternative to charging–discharging model, the multiple recombination center model was proposed in which structural changes in the NC geometry open and close pathways for trapping and non-radiative decay of charge carriers [302, 303]. These models also reproduce the power-law statistics of blinking. However, rapid non-radiative recombination is inconsistent with other experimental data, such as power-law delayed emission [240].

All existing models for blinking have one important weakness: they provide a mathematical description for blinking, but they lack a detailed microscopic (chemical) picture. In fact, it is surprising how little is established about blinking after 20 years of research, other than the statistics. A microscopic picture of blinking may eventually emerge from experiments combined with atomistic quantum mechanical calculations [264, 304] or from very challenging studies of correlated optical and time-resolved electron microscopy [305].

## Summary and Outlook

The last three decades have witnessed a remarkable development in the colloidal synthesis of composition-, size-, and shape-controlled semiconductor NCs and hetero-NCs, allowing researchers to make materials with tailored physical–chemical and optoelectronic properties by exploiting nanoscale phenomena, such as quantum confinement and surface effects. These effects, and their impact on the properties of semiconductor NCs and hetero-NCs, were discussed in detail in Sect. 2.

The availability of high-quality colloidal nanomaterials has in turn lead to great advances in the fundamental understanding of their properties. In this review, we focused on the excited-state dynamics in these nanomaterials, covering the whole range of relaxation processes spanning from the fs to the ms time scales: hot carrier relaxation (fs to ps), Auger decay of multi-carrier states (ps to ns), radiative decay (ns to μs), and photoluminescence intermittency (blinking), spectral diffusion, and delayed emission, which take place on time scales longer than ms. It is clear that the scientific community has a reasonably thorough understanding of many of the physical processes involved in the exciton formation and relaxation in semiconductor NCs and hetero-NCs, but there are still many poorly understood aspects and several knowledge gaps. As a result, a comprehensive theoretical framework capable of fully describing the exciton dynamics in semiconductor NCs and hetero-NCs has yet to emerge.

A particularly critical challenge is the understanding of the processes taking place at time scales longer than the radiative lifetime of the exciton, and the development of a detailed microscopic model that can relate these processes to chemical and structural transformations of the NC and/or its immediate vicinity. The understanding of the mechanisms underlying carrier trapping and photoluminescence quenching, and the role of capping ligands therein, is still fragmentary and merit a systematic and comprehensive investigation. Progress in this direction has been hampered by the lack of suitable tools, but many new techniques have appeared in recent years, and it is likely that further developments will make these issues amenable to experimental and computational investigation in the near future.

Another current limitation is that the large majority of the studies of the exciton dynamics in semiconductor NCs and hetero-NCs have been carried out on the prototypical case of CdSe and other Cd- and Pb-based compositions, while studies on emerging compositions such as InP, Cu chalcogenides and Si have been scarce. As a result, the latter class of nanomaterials is as yet poorly understood, despite their great potential as sustainable and less toxic alternatives to the conventional Cd- and Pb-based NCs and hetero-NCs. Recent advances in the synthesis of colloidal nanocrystals of these alternative compositions, and the growing interest that they have been attracting, will certainly lead to major efforts to close the gap in the understanding of their properties.
